# Ionizable lipids in bio-inspired nanocarriers

**DOI:** 10.1007/s00249-023-01633-4

**Published:** 2023-02-22

**Authors:** Vladimir P. Zhdanov

**Affiliations:** 1 Section of Nano and Biophysics, Department of Physics, Chalmers University of Technology, Göteborg, Sweden; 2 Boreskov Institute of Catalysis, Russian Academy of Sciences, Novosibirsk, Russia

**Keywords:** Nanoparticles, Ionizable lipids, Potential, Charge distribution, Langmuir–Stern equation, Poisson–Boltzmann equation

## Abstract

In applications of bio-inspired nanoparticles (NPs), their composition is often optimised by including ionizable lipids. I use a generic statistical model to describe the charge and potential distributions in lipid nanoparticles (LNPs) containing such lipids. The LNP structure is considered to contain the biophase regions separated by narrow interphase boundaries with water. Ionizable lipids are uniformly distributed at the biophase–water boundaries. The potential is there described at the mean-filed level combining the Langmuir–Stern equation for ionizable lipids and the Poisson–Boltzmann equation for other charges in water. The latter equation is used outside a LNP as well. With physiologically reasonable parameters, the model predicts the scale of the potential in a LNP to be rather low, smaller or about $k_\textrm{B}T/e$, and to change primarily near the LNP-solution interface or, more precisely, inside an NP near this interface because the charge of ionizable lipids becomes rapidly neutralized along the coordinate towards the center of a LNP. The extent of dissociation-mediated neutralization of ionizable lipids along this coordinate increases but only slightly. Thus, the neutralization is primarily due to the negative and positive ions related to the ionic strength in solution and located inside a LNP.

## Introduction

Nowadays, there are numerous efforts to use bio-inspired NPs in various biomedical applications [reviewed by Bost (2021), Hou et al. (2021), Mitchell et al. (2021), and Jackman et al. (2020); concerning the related physical aspects, see e.g. reviews by Lane (2020), Mendozza et al. (2019), and Zhdanov (2021)]. The best already commercialized example includes novel drugs and anti-viral vaccines based on RNA (mRNA or siRNA) delivery by LNPs (Hou et al. 2021). Typically, LNPs are fabricated by rapid mixing of an ethanol phase (lipid components) and an aqueous phase (mRNA molecules) under specific conditions, that is, pH and flow rate (Hou et al. 2021; Maeki et al. 2021). The size of LNPs is often in the range from 20–30 to 120 nm (Bost 2021). The decrease of size facilitates access to various locations in the body, whereas the size of $\sim 100$ nm is widely considered to be suitable in order to get a reasonable cell uptake (Bost 2021).

**Fig. 1:**
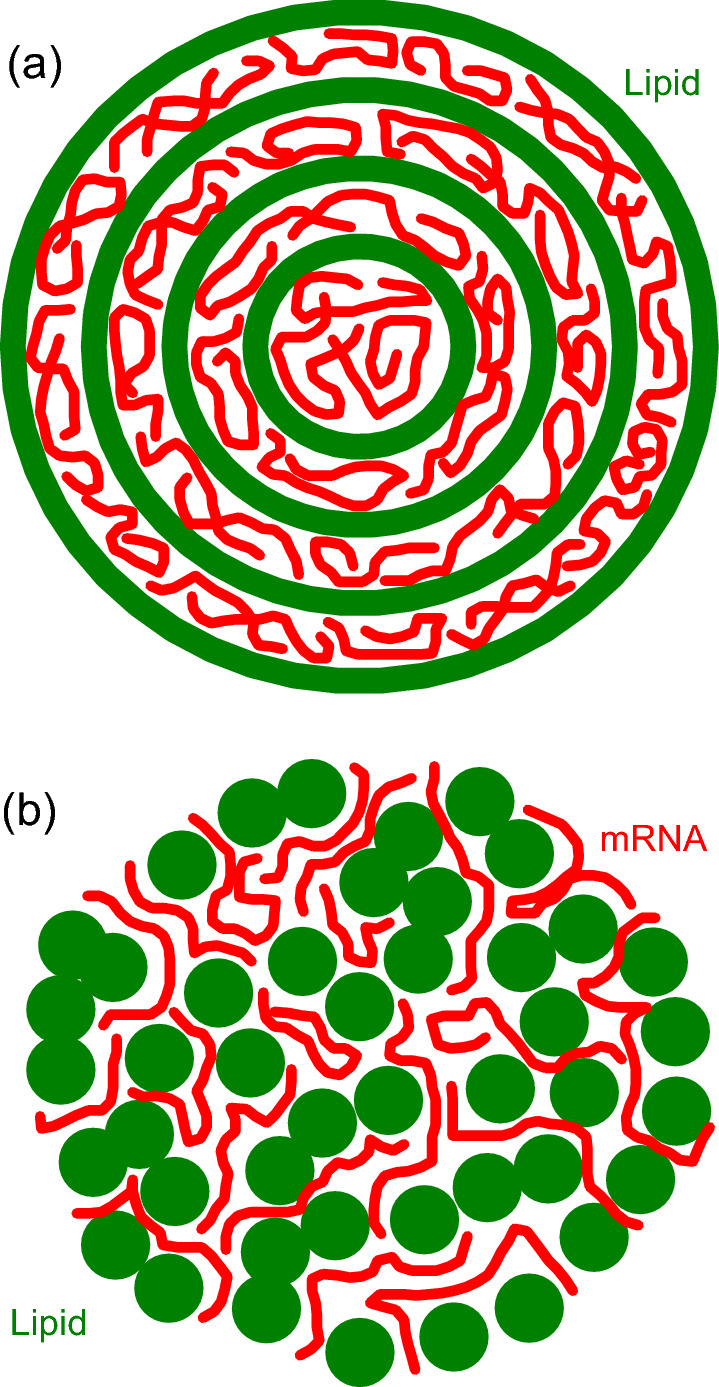
Schemes of **a** multilamellar and **b** nearly random LNP structures (Zhdanov 2017). In the analysis presented, the size of such LNPs is assumed to be in the range from 40 to 120 nm

The function of LNPs is often optimized by including ionizable lipids which are nearly neutral at physiological pH ($\simeq$ pH 7) and protonated, i.e., positively charged, at lower pH (Hou et al. 2021). The shape of LNPs of this category is close to spherical. Their structure is, however, typically complex and highly heterogeneous on the nm scale (schematically shown in Fig. 1). For example, the poorly ordered internal part of LNPs (i) can be reminiscent of multilamellar vesicles with solution and RNA located between lipid leaflets (Oberli 2017) or (ii) exhibit more complex and less ordered domains of “cubic” phase, formed by a lipid bilayer and containing water channels (Leung and Leal 2019) or (iii) “inverse hexagonal” phase (Arteta 2018). The thickness of water regions separating lipid-bilayer fragments in such structures is still poorly characterized. Roughly, one can consider that this thickness is about 3–4 nm. Many basic features of the NPs under consideration are expected to be close to those of biological membranes [the latter is extensively reviewed by Enkavi et al. (2017)]. Despite this similarity, the understanding of the physico-chemical basis underlying the NP function is now rather limited (Aliakbarinodehi 2022).

One of the aspects of the description of LNPs concerns the distribution of the corresponding potential inside and outside and extent of ionization of ionizable lipids inside. This aspect is of considerable intrinsic interest and is also important for quantification of the LNP-membrane interaction e.g. in the framework of the DLVO-type models (Ohshima 2012; Aliakbarinodehi 2022). To scrutinize the details of the potential under consideration, I recently used combination of the phenomenological Langmuir–Stern and Poisson–Boltzmann models with emphasis on ionizable lipids located on the external surface of NPs (Zhdanov 2022). What happens inside LNPs was not analyzed. In fact, the combination of these models allows one to describe various situations. Herein, I focus on the situation when the ionizable lipids are uniformly distributed inside a LNP. Mathematically, this case is opposed to that treated earlier (Zhdanov 2022). Taken together, the results obtained here and earlier allow one to form a view on the problem under consideration.

## Methods

In bio-inspired NPs (like LNPs), the biophase regions (e.g., the lipid phase) are separated by regions (of nanosized thickness) containing water and cargo. On the length scale above the lipid bilayer thickness (5 nm), an NP of this type can roughly be described at the coarse-grained level assuming the ionizable lipids to be uniformly distributed at the biophase-water boundaries and using there the locally averaged charge density and potential, $\rho$ and $\varphi$, and the effective dielectric permittivity, $\epsilon _\textrm{p}$ (the subscript “p” is associated with “particle”). This approach is applicable provided the NP size is appreciably larger than 5 nm. This condition is marginally satisfied for small LNPs (20–30 nm) and well satisfied for larger LNPs ($\ge 40$ nm). At this level, the potential inside a spherically symmetric NP is described as

1 $$\begin{aligned} \frac{d^2 \varphi }{dr^2}+\frac{2}{r}\frac{d \varphi }{dr} =-\frac{4\pi \rho }{\epsilon _\textrm{p}}, \end{aligned}$$

where $r\le R$ is the radial coordinate (*R* is the NP radius).

The charge density introduced above is formed by H$^+$ ions bound to ionizable lipids and negative and positive ions associated with the ionic strength of solution and located in relatively thin regions containing water. The way how these charges are interconnected depends on the extent of their penetration into an NP. Taking into account that the NPs under consideration are highly heterogeneous, their external interface is expected to have defects and/or channels, and accordingly H$^+$ ions and the negative and positive ions are likely to penetrate inside an NP and to be in equilibrium with those in solution outside NPs. The equations presented below imply this equilibrium.

In particular, according to the Langmuir–Stern model (reviewed by Koopal et al. 2020), the probability of ionization of an ionizable lipid is given by

2 $$\begin{aligned} p = c_\mathrm{H^+}/ [K_\textrm{a}\exp (e\varphi /k_\textrm{B}T) + c_\mathrm{H^+}], \end{aligned}$$

where *e* is the absolute value of the electron charge, $c_\mathrm{H^+}$ is the H$^+$ concentration in solution outside NPs, and $K_\textrm{a}$ is the H$^+$ attachment-detachment constant at $\varphi =0$. Employing this expression for ionizable lipids and the conventional Poisson–Boltzmann model for the negative and positive ions related to the ionic strength in solution (1:1 electrolyte) and located inside an NP (at $r\le R$), the charge density there can be represented as

3 $$\begin{aligned} \rho = \frac{e\chi c_* c_\mathrm{H^+}(1-f)}{K_\textrm{a}\exp (e\varphi /k_\textrm{B}T) + c_\mathrm{H^+}} - 2e c_{\circ } f \sinh \left( \frac{e\varphi }{k_\textrm{B}T} \right) , \end{aligned}$$

where *f* and $1-f$ is the fraction of the NP space occupied by water and biophase, respectively (the cargo is here considered to be negligible), $c_*$ is the lipid concentration in the biophase (to be specific, LNs are considered to be formed of lipid), $\chi$ is the fraction of ionizable lipids, and $c_{\circ }$ is the concentration of negative or positive ions responsible for the ionic strength in solution outside NPs.

With specification (3), the solution of Eq. (1) depends on the ratio of $c_\mathrm{H^+}$ and $K_\textrm{a}$ because $c_\mathrm{H^+}$ and $K_\textrm{a}$ are present only in the first term on the right-hand side of (3), and in fact this term depends on $c_\mathrm{H^+}/K_\textrm{a}$. Below, I operate with this ratio in order to keep the results as general as possible.

For solution outside an NP (at $r> R$), the Poisson–Boltzmann model yields

4 $$\begin{aligned} \frac{d^2 \varphi }{dr^2}+\frac{2}{r}\frac{d \varphi }{dr}= \frac{8\pi ec_{\circ }}{\epsilon _\textrm{s}} \sinh \left( \frac{e\varphi }{k_\textrm{B}T} \right) , \end{aligned}$$

where $\epsilon _\textrm{s}$ is the dielectric permittivity of solution.

The conventional boundary conditions for Eq. (1) at $r\rightarrow 0$, Eqs. (1) and (4) at $r\rightarrow R$, and Eq. and (4) at $r\rightarrow \infty$ are as follows

5 $$\begin{aligned}{} & {} \left. \frac{d \varphi }{dr}\right| _{r=0}=0, \end{aligned}$$

6 $$\begin{aligned}{} & {} \varphi \left| _{r=R-0}=\varphi \right| _{r=R+0}, \end{aligned}$$

7 $$\begin{aligned}{} & {} \epsilon _\textrm{p} \left. \frac{d \varphi }{dr}\right| _{r=R-0} = \epsilon _\textrm{s} \left. \frac{d \varphi }{dr}\right| _{r=R+0}, \end{aligned}$$

8 $$\begin{aligned}{} & {} \left. \varphi \right| _{r\rightarrow \infty }=0. \end{aligned}$$

Physically, condition (5) reflects the symmetry of the problem under consideration. Condition (6) is standard for the contact of dielectrics (Landau et al. 1984) and widely used for biological soft matter (lipid membranes, proteins, etc.) containing charges. Condition (7) describes the situation when the NP-solution interface does not contain the interfacial charge [as noticed in the Introduction, the case with this charge was analyzed elsewhere (Zhdanov 2022)]. Condition (8) reflects the neutrality of the whole system at $r\rightarrow \infty$.

The equations presented above are focused on ionizable lipids. In applications, LNPs contain RNA (mRNA or siRNA). In the context under consideration, the RNA role is twofold. First, the presence of RNA influences $$\epsilon _\textrm{p}$$, and this effect can be described implicitly using the effective value of $$\epsilon _\textrm{p}$$ (as it is done above and will be done below). Second, RNA contains (PO$$_2)^-$$ groups which may be not fully neutralized (Fingerhut 2021). The model presented does not take the latter into account, and this imposes limits on its applicability. In particular, the model is applicable provided the volume fraction occupied by RNA is not large and/or the (PO$$_2)^-$$ groups are primarily neutralized.

## Results

**Fig. 2:**
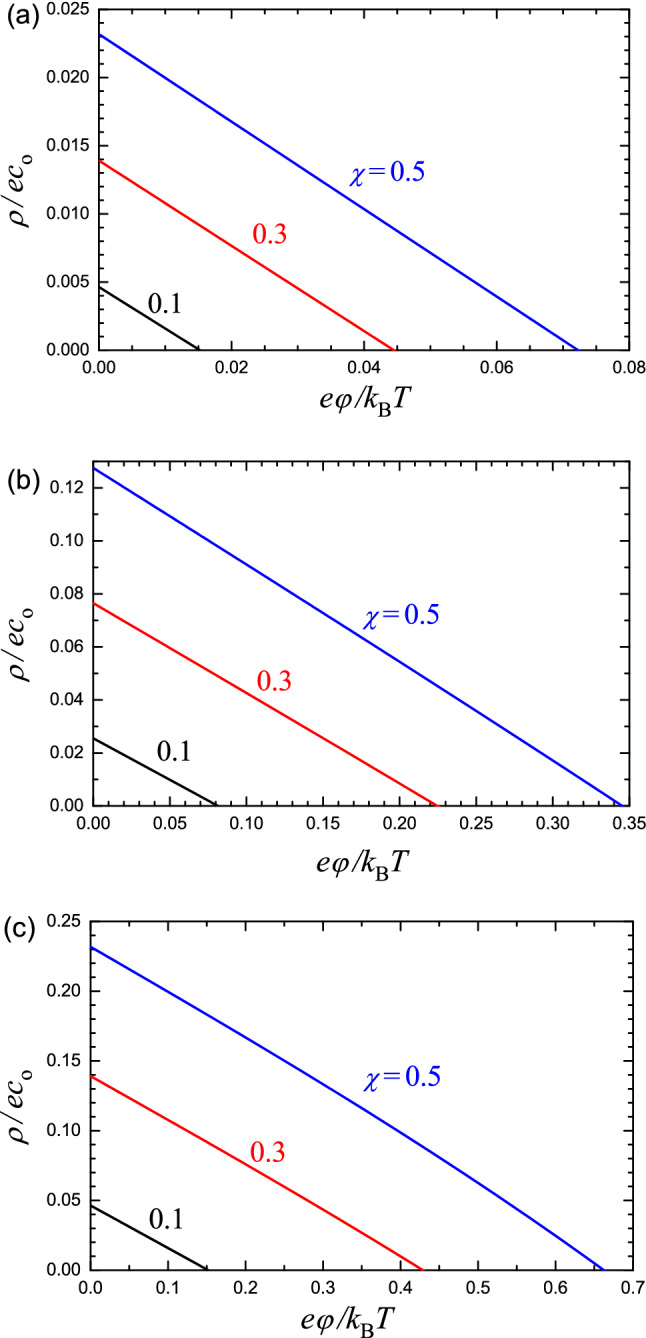
Normalized local density of charge in an NP as a function of $e\varphi /k_\textrm{B}T$ according to Eq. (3) for $c_\mathrm{H^+}/K_\textrm{a}=0.1$ **a**, 1 **b**, and 10 **c**; and $\chi =0.1$, 0.3, and 0.5. The other parameters are fixed as: $c_{\circ }=0.15\;\textrm{M}=10^{21}$ cm$^{-3}$, $c_*=0.6\times 10^{21}$ cm$^{-3}$, and $f=0.15$

Equations (1) and (4) contain many parameters and should in general be solved numerically. The full scale classification of the corresponding solutions is beyond my goals. In my analysis, I will slightly simplify these equations focusing on the biologically reasonable values of the parameters. To validate the simplifications, it is instructive to calculate $\rho$ as a function of $\varphi$ [Eq. (3)] at $0\le \varphi \le \varphi _*$, where $\varphi _*$ is the potential corresponding to $\rho =0$. This range of $\varphi$ describes the situations of interest ranging from that with negligible potential to that with full screening of the charge of ionizable lipids by negative and positive ions associated with the ionic strength of solution. The results of such calculations are shown in Fig. 2 for typical concentration of lipid molecules, $c_*=0.6\times 10^{21}$ cm$^{-3}$, physiological value of the electrolyte concentration, $c_{\circ }=0.15\;\textrm{M}=10^{21}$ cm$^{-3}$, $f=0.15$, $\chi =0.1$, 0.3, and 0.5, and a wide range of $c_\mathrm{H^+}/K_\textrm{a}$. The dependence of $\rho$ on $\varphi$ is seen to be very close to linear and accordingly can be represented as

9 $$\begin{aligned} \rho = \rho _{\circ }(1-\varphi /\varphi _*), \end{aligned}$$

where

10 $$\begin{aligned} \rho _{\circ } = e\chi c_* c_\mathrm{H^+}(1-f)/ (K_\textrm{a} + c_\mathrm{H^+}) \end{aligned}$$

is the charge density at $\varphi =0$ [cf. Equation (3)].

Substituting (9) into (1) yields

11 $$\begin{aligned} \frac{d^2 \varphi }{dr^2}+\frac{2}{r}\frac{d \varphi }{dr}= \frac{4\pi \rho _{\circ }(\varphi -\varphi _*)}{\epsilon _\textrm{p}\varphi _*}, \end{aligned}$$

The textbook solution of this equation at $0\le r\le R$ with condition (5) is

12 $$\begin{aligned} \varphi = \varphi _* -(\varphi _*- \varphi _{\circ }) \frac{R \sinh (r/\lambda _\textrm{p} )}{r \sinh (R /\lambda _\textrm{p})}, \end{aligned}$$

where $\varphi _{\circ }$ is the potential at $r=R$, and

13 $$\begin{aligned} \lambda _\textrm{p} =\left( \frac{\epsilon _\textrm{p}\varphi _*}{4\pi \rho _{\circ }}\right) ^{1/2} \end{aligned}$$

is the length scale determining the change of the potential in an NP near the external interface (for *r* close to *R*).

Taking into account that in solution (at $r\ge R$) $e\varphi /k_\textrm{B}T$ is low, the right-hand part of Eq. (4) can be linearized, i.e., Eq. (4) can be rewritten as

14 $$\begin{aligned} \frac{d^2 \varphi }{dr^2}+\frac{2}{r}\frac{d \varphi }{dr}= \frac{8\pi e^2c_{\circ }\varphi }{\epsilon _\textrm{s}k_\textrm{B}T}. \end{aligned}$$

The conventional solution of the latter equation with conditions (6) and (8) is

15 $$\begin{aligned} \varphi =\varphi _{\circ }\frac{R }{r} \exp [-(r-R)/\lambda _\textrm{p}], \end{aligned}$$

where

16 $$\begin{aligned} \lambda _\textrm{s} =\left( \frac{\epsilon _\textrm{s}k_\textrm{B}T}{8\pi e^2c_{\circ }}\right) ^{1/2} \end{aligned}$$

is the Debye length determining the change of the potential at $r\ge R$.

The value of $\varphi _{\circ }$ in expressions (12) and (15) is determined by condition (7). With these expressions, this condition is somewhat cumbersome. In practically important situations, it can, however, be simplified. From this perspective, it is instructive to recall that under physiological conditions, the scale of $\lambda _\textrm{s}$ is known to be about 1 nm. The ratio of $\lambda _\textrm{p}$ and $\lambda _\textrm{s}$ is

17 $$\begin{aligned} \frac{\lambda _\textrm{p}}{\lambda _\textrm{s}} = \left( \frac{2\epsilon _\textrm{p}c_{\circ } e^2\varphi _*}{\epsilon _\textrm{s} \rho _{\circ }k_\textrm{B}T}\right) ^{1/2}. \end{aligned}$$

In this expression, the ratio $\epsilon _\textrm{p}/\epsilon _\textrm{s}$ is appreciably smaller than unity, whereas the ratio $2c_{\circ } e^2\varphi _*/\rho _{\circ }k_\textrm{B}T$ is often appreciably larger than unity, so that the scales of $\lambda _\textrm{p}$ and $\lambda _\textrm{s}$ are comparable, i.e., $\lambda _\textrm{p}$ is also about 1 nm, whereas the scale of *R* is much larger, from 10–15 nm for small LNPs to $\sim$30–50 nm for more conventional LNPs. Practically, this means that the charge and potential change primarily near the interface at $|r-R|\le 3$–5 nm. In this region, the ratio *R*/*r* in expressions (12) and (15) is close to unity and can be dropped. In addition, the hyperbolic sines can in (12) be replaced by an exponential function. It can be safely done at least for LNPs with a size of $\sim$60–100 nm. With these simplifications, expressions (12) and (15) can be rewritten as

18 $$\begin{aligned} \varphi = \varphi _* -(\varphi _*- \varphi _{\circ }) \exp [-(R-r)/\lambda _{p}], \end{aligned}$$

19 $$\begin{aligned} \varphi =\varphi _{\circ }\exp [-(r-R)/\lambda _{s}], \end{aligned}$$

and then condition (7) yields

20 $$\begin{aligned} \frac{\epsilon _\textrm{p}(\varphi _*- \varphi _{\circ })}{\lambda _\textrm{p}}= \frac{\epsilon _\textrm{s}\varphi _{\circ }}{\lambda _\textrm{s}},\;\;\textrm{or}\;\; \varphi _{\circ } =\frac{\epsilon _\textrm{p}\lambda _\textrm{s}\varphi _*}{\epsilon _\textrm{p}\lambda _{s}+\epsilon _\textrm{s}\lambda _\textrm{p}}. \end{aligned}$$

For solution (water) outside an NP, we have $\epsilon _\textrm{s}=80$. Inside an NP, $\epsilon _\textrm{p}$ is formed primarily by biophase (lipid and cargo) with $\epsilon = 3-5$ and partly by water-containing regions (with $f \simeq 0.15$). The average permittivity, $\epsilon _\textrm{p}$, can in this case be calculated by using one of the numerous available approximations [see, e.g., the review by Sarami et al. (2019) and references therein]. For $f \simeq 0.15$, the scale of $\epsilon _\textrm{p}$ is 7–9.

**Fig. 3:**
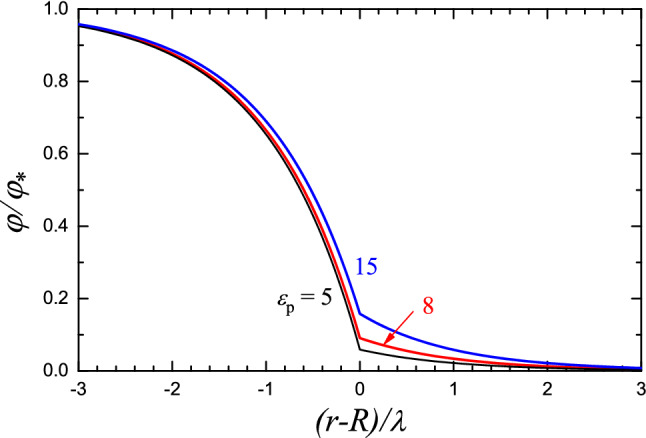
Potential profiles near the NP-solution interface according to Eqs. (18)–(20) with $\lambda _\textrm{p}=\lambda _\textrm{s}=\lambda$, $\epsilon _\textrm{s}=80$, and $\epsilon _\textrm{p}=5$, 8, and 18

**Fig. 4:**
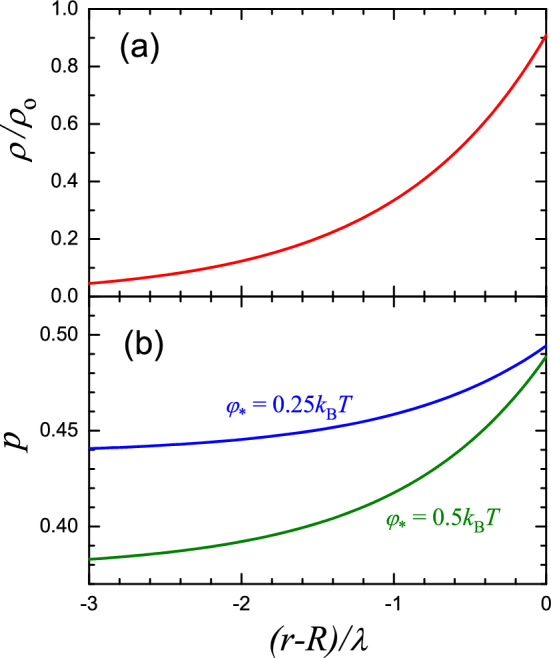
**a** Charge density inside an NP near the NP-solution interface according to Eq. (9), and** b** probability of ionization of an ionizable lipid according to Eq. (2) with $c_\mathrm{H^+}/K_\textrm{a}=1$ and $\varphi _*/k_\textrm{B}T =0.25$ and 0.5 [cf. e.g. Figure 2(c)]. The results were obtained using the potential defined by Eq. (18) and calculated with $\epsilon _\textrm{p}=8$ (as shown in Fig. 3)

A typical potential profile calculated near the NP-solution interface using expressions (18)-(20) with $\lambda _\textrm{p}=\lambda _\textrm{s}$, $\epsilon _\textrm{p}=8$, and $\epsilon _\textrm{s}=80$ is shown in Fig. 3. The corresponding drop of the charge density in an NP in the inward direction is exhibited in Fig. 4(a) together with the probability of ionization of an ionizable lipid [Fig. 4(b)].

The estimate of $\epsilon _\textrm{p}$ above is obviously not very accurate. In reality, $\epsilon _\textrm{p}$ can be somewhat smaller or larger than 8. To illustrate the role of this factor or, in other words, the sensitivity of the results with respect to the value of $\epsilon _\textrm{p}$, I have calculated the potential profile also for $\epsilon _\textrm{p}=5$ and 15 (Fig. 3). One can see that this effect of variation of the value of $\epsilon _\textrm{p}$ on the results is modest.

In applications, only a small amount of LNPs are able to escape from the endosomes, and this process is considered to be crucial for effective RNA delivery (Hou et al. 2021). To optimize electrostatic interaction and fusion with negatively charged endosomal membranes, resulting in the leak of RNA molecules into the cytoplasm, the ionizable lipids are often chosen so that the pH level corresponding to $p=1/2$ [in Eq. (2) with $\varphi =0$] is roughly equal to the endosomal level, pH 5–6 (Hou et al. 2021; Bost 2021). This condition ($p=1/2$) corresponds to $c_\mathrm{H^+}/K_\textrm{a}=1$. This means that the results obtained with $c_\mathrm{H^+}/K_\textrm{a}=1$ [Figs. 2(b), 3, and 4] represent the practically interesting situations with pH 5–6.

## Discussion and conclusion

The results obtained (Figs. 2–4) allow me to draw the following conclusions:

(i) The scale of the potential related to the presence of ionizable lipids in an NP is rather low, smaller or about $k_\textrm{B}T/e$ (Fig. 2).
(ii) The potential changes primarily near the NP-solution interface or, more precisely, inside an NP near this interface (Fig. 3).
(iii) Feature (ii) above means that the charge of ionizable lipids becomes rapidly neutralized along the coordinate towards the center of an NP [Fig. 4a]. In this context, it is of interest that the extent of neutralization of ionizable lipids along this coordinate due to detachment of H$^+$ increases but only slightly [Fig. 4b]. Thus, the predicted neutralization [Fig. 4(a)] is primarily due to the negative and positive ions related to the ionic strength in solution and located inside an NP.

As already noticed in the introduction, the model proposed here is opposite compared to that treated earlier (Zhdanov 2022). The two corresponding conclusions [items (i) and (ii) above] are, however, similar to those drawn earlier (Zhdanov 2022). This means that the specifics of the problem under consideration is not too sensitive to the details of its description. In fact, the models proposed here and earlier (Zhdanov 2022) are complimentary and support each other.

The conditions of the applicability of the model proposed have already been discussed during the introduction and/or derivation of the key equations. The theoretical results presented can be, however, be debated from various perspectives and/or readily generalized in various directions. A few related remarks are as follows.

For example, the Poisson–Boltzmann equation should be corrected at high concentration of ions in solution (Härtel 2017). Under physiological conditions, such corrections are, however, often not crucial.

Inhomogeneous and/or nonlocal water permittivity in the vicinity of interfaces and ions is another issue (Loche et al. 2020; Vatin et al. 2021). The current model takes this effect into account only to some extent using the effective permittivity inside an NP. Outside an NP, the water permittivity is considered to be the same as in the bulk. In electrochemistry and some other areas of natural sciences, the latter approximation was long recognized to be not always accurate. A good example where the deviations are appreciable is water near or between hydrophobic graphene layers [the corresponding experimental and theoretical studies were performed by Fumagalli et al. (2018) and Monet et al. (2021), respectively]. Such layers are incompatible with water, and, as expected, the effect of their presence on local arrangement and dielectric properties of water is large. In contrast, I discuss the system where the interface is formed by water and hydrophilic heads of lipids. The hydrophilicity of these heads itself is in favour of their low effect on local arrangement and dielectric properties of water. An additional factor favourable for this conclusion is that the heads of lipids are well known to not close packed. The lateral distance between the heads is sufficient for penetration of water molecules [see, e.g., Fig. 28(B) in the review by Enkavi et al. (2019)], i.e., the water molecules, located near the interface at the solution side, contact not only lipid heads but also water molecules located near the interface at the lipid-bilayer side. For these reasons, the dielectric permittivity of water near the interface is expected to be closer to that in the bulk and/or the region where the deviations are appreciable is expected to be narrower [the latter is in agreement with the molecular dynamics simulations presented by Bonthuis et al. (2012)]. In particular, the bulk permittivity is widely used e.g. to describe water near a lipid bilayer in numerous models of ion channels in lipid bilayers.

Changes of the Born solvation energy near interfaces can also be mentioned [this effect is discussed e.g. by Liu and Lu (2017)]. At the simplest level, it can be done by multiplying $c_{\circ }$ in the Poisson–Boltzmann equation by the corresponding factor or, alternatively, by redefining $c_{\circ }$.

Some other corrections can be discussed and/or introduced into the model as well.

## Data Availability

All the data are given in the article.

## References

[CR1] Aliakbarinodehi N (2022). Interaction kinetics of individual mRNA-containing lipid nanoparticles with an endosomal membrane mimic: dependence on pH, protein corona formation, and lipoprotein depletion. ACS Nano.

[CR2] Arteta NY (2018). Successful reprogramming of cellular protein production through mRNA delivered by functionalized lipid nanoparticles. Proc Natl Acad Sci USA.

[CR3] Bonthuis DJ, Gekle S, Netz RR (2012). Profile of the static permittivity tensor of water at interfaces: consequences for capacitance, hydration interaction and ion adsorption. Langmuir.

[CR4] Bost JP (2021). Delivery of oligonucleotide therapeutics: chemical modifications, lipid nanoparticles, and extracellular vesicles. ACS Nano.

[CR5] Enkavi G, Javanainen M, Kulig W, Rog T, Vattulainen I (2019). Multiscale simulations of biological embranes: the challenge to understand biological phenomena in a living substance. Chem Rev.

[CR6] Fingerhut BP (2021). The mutual interactions of RNA, counterions and water - quantifying the electrostatics at the phosphate-water interface. Chem Commun.

[CR7] Fumagalli F (2018). Anomalously low dielectric constant of confined water. Science.

[CR8] Härtel A (2017). Structure of electric double layers in capacitive systems and to what extent (classical) density functional theory describes it. J Phys Condens Matter.

[CR9] Hou X, Zaks T, Langer R, Dong Y (2021). Lipid Nanoparticles for mRNA delivery. Nature Rev Mater.

[CR10] Jackman JA (2020). Biomimetic nanomaterial strategies for virus targeting: antiviral therapies and vaccines. Adv Funct Mater.

[CR11] Koopal L, Tan W, Avena M (2020). Equilibrium mono- and multicomponent adsorption models: from homogeneous ideal to heterogeneous non-ideal binding. Adv Coll Interf Sci.

[CR12] Landau LD, Lifshitz EM, Pitaevskii LP (1984). Electrodynamics of Continuous Media.

[CR13] Lane LA (2020). Physics in nanomedicine: phenomena governing the in vivo performance of nanoparticles. Appl Phys Rev.

[CR14] Leung SSW, Leal C (2019). The stabilization of primitive bicontinuous cubic phases with tunable swelling over a wide composition range. Soft Matter.

[CR15] Liu X, Lu B (2017). Incorporating Born solvation energy into the three-dimensional Poisson-Nernst-Planck model to study ion selectivity in KcsA K$$^+$$ channels. Phys Rev E.

[CR16] Loche P, Ayaz C, Wolde-Kidan A, Schlaich A, Netz RR (2020). Universal and nonuniversal aspects of electrostatics in aqueous nanoconfinement. J Phys Chem B.

[CR18] Maeki M, Uno S, Niwa A, Okada Y, Tokeshi M (2021). Microfluidic technologies and devices for lipid nanoparticle-based RNA delivery. J Contr Release.

[CR17] Mendozza M, Caselli L, Salvatore A, Montis C, Berti D (2019). Nanoparticles and organized lipid assemblies: from interaction to design of hybrid soft devices. Soft Matt.

[CR19] Mitchell MJ, Billingsley MM, Haley RM, Wechsler ME, Peppas NA, Langer R (2021). Engineering precision nanoparticles for drug delivery. Nature Rev Drug Discov.

[CR20] Monet G, Bresme F, Kornyshev A, Berthoumieux H (2021). Nonlocal dielectric response of water in nanoconfinement. Phys Rev Lett.

[CR21] Ohshima H, Ohshima H (2012). Fundamentals. Electrical Phenomena at Interfaces and Biointerfaces Fundamentals and Applications in Nano- Bio- and Environmental Sciences.

[CR22] Oberli MA (2017). Lipid nanoparticle assisted mRNA delivery for potent cancer immunotherapy. Nano Lett.

[CR23] Sarami MA, Moghadam M, Gilani AG (2019). Modified dielectric permittivity models for binary liquid mixture. J. Molec. Liq..

[CR25] Vatin M, Porro A, Sator N, Dufrêche J-F, Berthoumieux H (2021). Electrostatic interactions in water: a nonlocal electrostatic approach. Molec Phys.

[CR26] Zhdanov VP (2017). Kinetics of lipid-nanoparticle-mediated intracellular mRNA delivery and function. Phys Rev E.

[CR24] Zhdanov VP (2021). Virology from the perspective of theoretical colloid and interface science. Curr Opin Coll Interf Sci.

[CR27] Zhdanov VP (2022). Lipid nanoparticles with ionizable lipids: statistical aspects. Phys Rev E.

